# Phenotypically distinct anti-insulin B cells repopulate pancreatic islets after anti-CD20 treatment in NOD mice

**DOI:** 10.1007/s00125-019-04974-y

**Published:** 2019-08-23

**Authors:** Joanne Boldison, Larissa C. Da Rosa, Lucy Buckingham, Joanne Davies, Li Wen, F. Susan Wong

**Affiliations:** 1 Division of Infection and Immunity, Cardiff University School of Medicine, Cardiff, CF14 4XN UK; 2 Section of Endocrinology, School of Medicine, Yale University, New Haven, CT USA

**Keywords:** Anti-CD20 treatment, Anti-insulin B cells, Type 1 diabetes

## Abstract

**Aims/hypothesis:**

Autoreactive B cells escape immune tolerance and contribute to the pathogenesis of type 1 diabetes. While global B cell depletion is a successful therapy for autoimmune disease, the fate of autoreactive cells during this treatment in autoimmune diabetes is unknown. We aimed to identify and track anti-insulin B cells in pancreatic islets and understand their repopulation after anti-CD20 treatment.

**Methods:**

We generated a double transgenic system, the VH125.hCD20/NOD mouse. The VH125 transgenic mouse, expressing an increased frequency of anti-insulin B cells, was crossed with a human CD20 (hCD20) transgenic mouse, to facilitate B cell depletion using anti-CD20. B cells were analysed using multiparameter and ImageStream flow cytometry.

**Results:**

We demonstrated that anti-insulin B cells were recruited to the pancreas during disease progression in VH125.hCD20/NOD mice. We identified two distinct populations of anti-insulin B cells in pancreatic islets, based on CD19 expression, with both populations enriched in the CD138^int^ fraction. Anti-insulin B cells were not identified in the plasma-cell CD138^hi^ fraction, which also expressed the transcription factor Blimp-1. After anti-CD20 treatment, anti-insulin B cells repopulated the pancreatic islets earlier than non-specific B cells. Importantly, we observed that a CD138^int^insulin^+^CD19^−^ population was particularly enriched after B cell depletion, possibly contributing to the persistence of disease still observed in some mice after anti-CD20 treatment.

**Conclusions/interpretation:**

Our observations may indicate why the loss of C-peptide is only temporarily delayed following anti-CD20 treatment in human type 1 diabetes.

**Electronic supplementary material:**

The online version of this article (10.1007/s00125-019-04974-y) contains peer-reviewed but unedited supplementary material, which is available to authorised users.

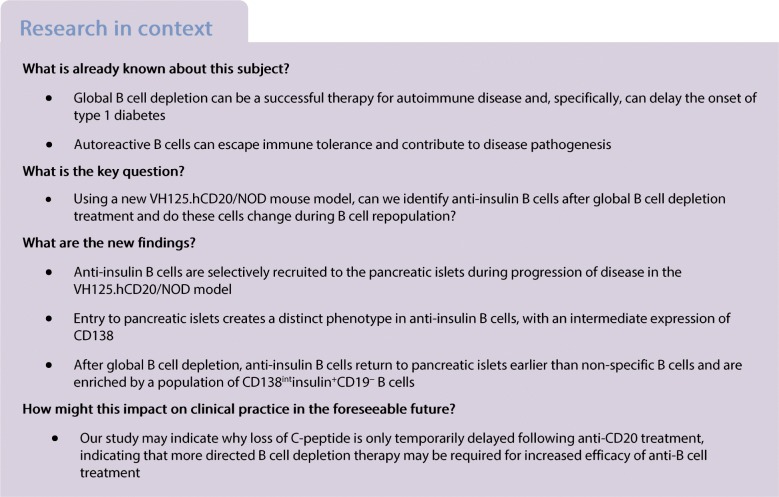



## Introduction

Type 1 diabetes is an organ-specific autoimmune disease characterised by immune-mediated beta cell destruction in pancreatic islets, which results in deficient insulin production. Although T cells directly damage insulin-producing beta cells, B cells are key in this multifactorial process. Rituximab treatment (B cell depletion therapy) in individuals with type 1 diabetes delayed loss of C-peptide in the first year after diagnosis [1]. Furthermore, B cell depletion in NOD mice restored normoglycaemia in a proportion of diabetic mice [2, 3] and improved islet allograft survival rate [4].

However, global B cell depletion may induce unwanted side effects [5] and specific B cell targeting may be safer. In this context, rituximab specifically suppressed anti-insulin autoantibodies more than other autoantibodies [6]. Animal studies have shown anti-insulin B cells are important in the pathogenesis of type 1 diabetes. A B cell transgenic mouse, expressing a fixed heavy-chain B cell receptor (BCR) with potential for insulin binding, had an increased frequency of anti-insulin B cells in a polyclonal repertoire (VH125) and accelerated diabetes onset [7]. Conversely, the control heavy-chain transgenic mouse (VH281), with restricted insulin binding, had a reduced diabetes incidence [7]. Furthermore, anti-insulin B cell depletion using the mAb123 antibody in NOD mice protected against spontaneous diabetes [8]. Thus, autoreactive anti-insulin B cells play a particularly important role in the pathogenesis of type 1 diabetes.

Although self-reactive B cells undergo central tolerance in the bone marrow, autoantigen-specific B cells are found in the peripheral B cell repertoire, in a functionally silent or anergic state [9]. Anti-insulin B cells (125Tg model) have impaired responses to both innate and adaptive B cell stimulators such as lipopolysaccharide and anti-CD40, respectively [10]. However, these B cells can still present antigen and stimulate both naive and insulin-specific CD4 T cells [11], suggesting that even in an impaired state, autoreactive B cells can still promote type 1 diabetes.

B1 B cells are important players in initiation of disease [12] and are present early in the pancreas of NOD [12, 13] and DO11xRIP-mOVA mice [12–14], whereas established islet B cells have a more follicular phenotype [14], although antigen specificity has not been investigated. Anti-insulin B cells have been clearly identified in pancreatic islets of NOD mice and are enriched at this site, compared with secondary lymphoid organs [15]. The aim of our study was to track the fate of anti-insulin B cells in the pancreatic islets after anti-CD20 treatment by using a double transgenic NOD mouse. Here, we have focused specifically on the phenotype and functionality of insulin-binding cells in the pancreatic islets, a tissue that is not accessible in humans at specific times in the pathogenesis of diabetes.

## Methods

### Mice

NOD/Caj mice, originally from Yale University, were bred in-house at Cardiff University. VH125/NOD B cell transgenic mice [NOD.Cg-Tg(*Igh*-6/*Igh*-V125)2Jwt/JwtJ] and VK125/NOD B cell transgenic mice [NOD.Cg-Tg(*Igk*-C/*Igk*-V1251)Jwt/JwtJ] were purchased from the Jackson Laboratory [7]. Mice were inter-crossed to generate 125Tg mice. Human CD20 (hCD20) transgenic mice (BALB/c background), were backcrossed to NOD mice more than ten generations, and designated hCD20/NOD mice [2]. These mice were crossed with VH125/NOD B cell transgenic mice to generate VH125.hCD20/NOD mice. The G9*Cα*^*−/−*^NOD mice [16] were bred in-house at Cardiff University. All mice received water and irradiated food ad libitum and were housed in specific-pathogen-free isolators or scantainers, with a 12 h dark–light cycle, at Cardiff University. All animal experiments were approved by Cardiff University ethical review process and conducted under UK Home Office licence in accordance with the UK Animals (Scientific Procedures) Act, 1986 and associated guidelines.

### Cell preparations

Pancreatic lymph nodes (PLNs) were disrupted mechanically with a 30G needle. Bone marrow cells were flushed from the hind femur and tibia. Spleens were homogenised and erythrocytes were lysed. Pancreases were inflated with collagenase P solution (Roche, Welwyn Garden City, UK) in HBSS via the common bile duct, followed by collagenase digestion with shaking at 37°C for 10 min. Islets were isolated by Histopaque density centrifugation (Sigma-Aldrich, Dorset, UK), hand-picked under a dissecting microscope and then trypsinised to generate a single-cell suspension. Islet cells were rested at 37°C 5% CO_2_ in complete Iscove Modified Dulbecco’s Medium (IMDM) overnight.

### Anti-insulin B cell detection

Anti-insulin B cells were detected using recombinant human insulin–FITC (Sigma-Aldrich). For flow cytometric analysis, B cells were incubated with 1.25 μg/ml insulin–FITC, together with surface receptor antibodies, as described below. To ensure specific insulin binding, a competitive assay was performed alongside experimental tubes using non-labelled insulin (Sigma-Aldrich) at 25 μg/ml (20×). For detection of islet anti-insulin B cells, the cells were incubated with insulin–FITC for 1 h at 37°C.

### Flow cytometry

Cells were incubated with TruStain (anti-mouse CD16/32; Biolegend, London, UK) for 10 min at 4°C, followed by fluorochrome-conjugated monoclonal antibodies (mAbs) against cell surface markers for 30 min at 4°C. Multivariable flow cytometry was carried out using mAbs: CD3 (145-2C11), B220 (RA3-6B2), IgD (11-26c.2a), CD138 (281-2), CD86 (PO3), CD80 (16-10A1), CD11c (N418), CD11b (M1/70), CD5 (53-7.3), hCD20 (2H7) and mCD20 (SA271G2) purchased from Biolegend; CD21 (7G6), CD69 (H1.2F3), CD43 (S7) and IgM^a^ (DS-1) purchased from BD Biosciences (Wokingham, UK); CD19 (eBio1D3) and CD23 (B3B4) purchased from eBioscience (San Diego, CA, USA) and CD220 (FAB1544P) purchased from R&D systems (Abingdon, UK). Dead cells were excluded from analysis by Live/Dead exclusion dye (Invitrogen, Paisley, UK). For intracellular cytokine staining, cells were stimulated with phorbal 12-myristrate-13-acetate (PMA) (50 ng/ml), ionomycin (500 ng/ml) and monensin (3 μg/ml) (all from Sigma-Aldrich) for 3 h. After extracellular staining, cells were fixed using a fixation/permeabilisation kit (BD Biosciences) according to manufacturer’s instructions and subsequently stained for intracellular cytokines (IFNγ [XMG1.2], Biolegend) or with appropriate isotype controls. For Blimp-1 (5E7; BD Biosciences) staining, cells were fixed/permeabilised using eBioscience nuclear transcription kit. Cell suspensions were acquired on an LSRFortessa (FACSDIVA software, BD Biosciences) and analysed using Flowjo software, version 10.1 (Tree Star, Ashland, OR, USA).

### Anti-CD20 treatment

Female VH125.hCD20/NOD mice, 6–8 weeks of age were chosen at random to receive either anti-hCD20 antibody (clone 2H7; Bio-XCell, West Lebanon, NH, USA) or control IgG2b antibody (clone MPC-11; Bio-XCell [2, 17, 18]), as described previously [19]. The treatment comprised four injections at intervals of 3 days, each containing 500 μg of antibody in 200 μl of saline (NaCl 154 mmol/l), the first of which was i.v., followed by three i.p. injections.

### Functional assay

Anti-insulin B cells from VH125.hCD20/NOD mice were labelled with insulin–FITC conjugate and enriched using FITC microbeads (Miltenyi Biotec, Bisley, UK), according to manufacturer’s instructions. The purity of the enriched fraction ranged from 25–45% insulin binding. B cells from both the insulin-negative and insulin-positive fraction were stimulated with 5 μg/ml anti-CD40 (Bio-Xcell) for 24 h. Splenic CD8^+^ T cells from G9*Cα*^−/−^ mice were prepared using CD8^+^ T cell isolation kits (Miltenyi Biotec) and cultured with B cell fractions (1:3 ratio). After 24 h, activation markers were examined by flow cytometry.

### Imagestream analysis

Single cells from rested islets or splenocytes were surface-stained and fixed as described above. Fluorescent images were collected using INSPIRE software on an Amnis Imagestream (Merck, Kenilworth, NJ, USA) imaging flow cytometer, with a minimum of 50,000 cells per sample, and analysed using Amnis IDEAS software (Merck).

### Statistics

Statistical analyses were performed using GraphPad Prism (GraphPad Software, San Diego, CA, USA). Significance was determined by one-way ANOVA followed by a Dunn’s multiple comparison, or two-way ANOVA followed by a Bonferroni post hoc test for more than two variables and a Mann–Whitney *U* test for only two variables. Data were significant at *p* < 0.05.

## Results

### Characterisation of anti-insulin B cells in VH125.hCD20/NOD mice

VH125/NOD transgenic mice expressing an increased frequency of anti-insulin B cells [7] were crossed with hCD20/NOD mice [2] to generate VH125.hCD20/NOD mice, facilitating the depletion of B cells using the 2H7 mAb (depletes B cells expressing human CD20). Expression of single or double transgenes did not affect T and B cell development (ESM Fig. 1a,b). However, we noted an increase in splenic marginal zone B cells when the VH125 transgene was present, as previously reported [20] (ESM Fig. 1c).

**Fig. 1:**
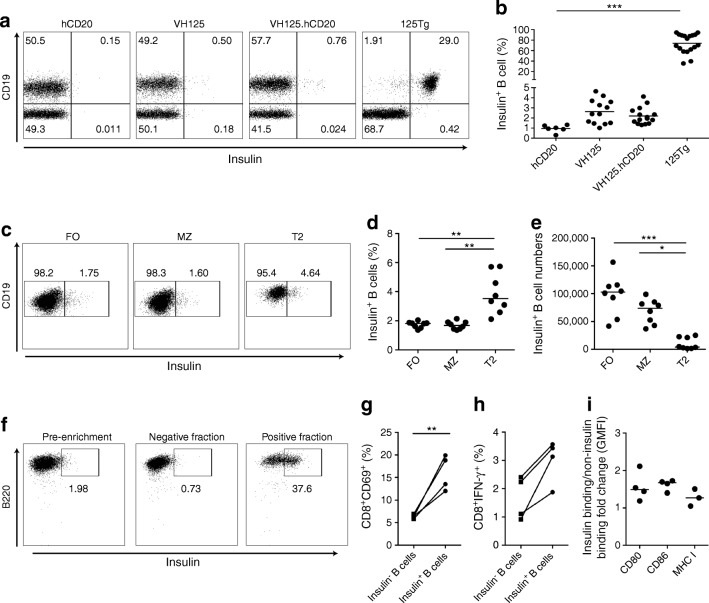
Anti-insulin B cells successfully present antigen to insulin-specific CD8 T cells. (**a**, **b**) Anti-insulin B cells from spleens of hCD20/NOD (hCD20), VH125, VH125.hCD20/NOD (VH125.hCD20) and 125Tg mice analysed by flow cytometry using human insulin conjugated to FITC fluorochrome. Representative flow cytometric plots (**a**) and quantification of total frequency of insulin-positive B cells (**b**) in each transgenic strain are shown. *n* = 6 (hCD20/NOD); *n* = 13 (VH125); *n* = 15 (VH125.hCD20/NOD); *n* = 18 (125Tg). Cells were gated on live CD3^−^CD19^+^ cells. (**c**–**e**) VH125.hCD20/NOD splenic B cells were labelled for compartments (follicular zone [CD23^hi^CD21^lo^], marginal zone [CD23^lo^CD21^hi^], transitional 2 cells [CD23^hi^CD21^hi^]) and counterstained with insulin–FITC (*n* = 8 mice). Representative flow cytometric plots of insulin^+^ B cells in each compartment (**c**), quantification of the percentage of B cells (**d**) and quantification of absolute insulin^+^ B cell numbers (**e**) are shown. (**f**–**i**) Anti-insulin B cells from spleens of groups (*n* = 3–5 pooled) of VH125.hCD20/NOD mice were enriched using insulin–FITC labelling and FITC microbeads and stimulated with 5 mg/ml anti-CD40 for 24 h before co-culture with purified G9*Cα*^−/−^ insulin-specific CD8 T cells. CD8 T cells were then analysed for activation markers after 24 h. Positive insulin fractions after enrichment ranged from 25% to 45% (*n* = 4 groups). Representative plots showing enrichment of insulin-binding B cells by anti-FITC microbeads, labelled with B220 (as B220-based enrichment) and insulin (**f**) and the percentage of CD69 (**g**) and IFN-γ (**h**) expression on live CD8 T cells after culture with insulin-positive and -negative fractions are shown. (**i**) Fold change in GMFI of co-stimulation markers CD80 and CD86 and MHC I on insulin-positive and -negative B cells (live CD19^+^) after co-culture. All data shown are representative of at least three independent experiments. Horizontal lines represent median values. *p < 0.05, **p < 0.01 and ***p < 0.001 (one-way ANOVA) in (**b**, **d**, **e**). **p < 0.01 (Mann–Whitney *U* test) in (**g**). FO, follicular zone; MZ, marginal zone; T2, transitional 2

We examined anti-insulin B cells using FITC-conjugated insulin in four transgenic mouse strains (hCD20/NOD, VH125 and VH125.hCD20/NOD mice and 125Tg mice [transgenic for both heavy (VH) and light (VL) chains of the 125 insulin-specific BCR] as a positive control [21]). We demonstrated an increased percentage of anti-insulin B cells in the spleen of both VH125 and VH125.hCD20/NOD mice compared with hCD20.NOD mice (Fig. 1a,b). Anti-insulin B cells populating all splenic compartments in the VH125.hCD20/NOD mice (Fig. 1c–e), were comparable with anti-insulin B cells that escape central tolerance in the spleen in VH125 mice [8]. Anti-insulin B cells were proportionally enriched in the T2 population (CD21^hi^CD23^hi^) (Fig. 1d), which plays a role in antigen-specific positive selection as these cells proliferate upon BCR engagement [22]. The absolute number of anti-insulin B cells was significantly higher in both follicular and marginal zones (Fig. 1e), sites where BCR signalling is fundamental for B cell maturity [23].

### Anti-insulin B cells can successfully present antigen to insulin-specific CD8^+^ T cells

To assess functionality, we investigated whether anti-insulin B cells could present insulin to and activate insulin-specific CD8^+^ T cells from the monoclonal G9*Cα*^−/−^ CD8^+^TCR transgenic mouse. To obtain sufficient anti-insulin B cells, we used a two-step enrichment approach of insulin–FITC detection and anti-FITC microbeads (Fig. 1f). We obtained approximately 20 times more anti-insulin B cells (Fig. 1f) compared with the multi-step biotinylated antibody technique used by Smith et al [24] whereby enrichment was approximately 8%. Insulin-positive and insulin-negative fractions were then cultured with G9*Cα*^−/−^ CD8^+^ T cells for 24 h. Significant CD69 upregulation (*p* = 0.026) and increased IFN-γ (*p* = 0.114, not statistically significant) production from CD8^+^ T cells was observed, when cultured with insulin-positive B cells compared with insulin-negative B cells (Fig. 1g,h). Insulin-positive B cells cultured with G9*Cα*^−/−^ CD8^+^ T cells expressed increased CD86 (geometric mean fluorescent intensity [GMFI], *p* = 0.0571) when compared with insulin-negative B cells (Fig. 1i). We also observed a non-significant increase in the GMFI of CD80 and MHC I (Fig. 1i). Thus, anti-insulin B cells can successfully present to and activate IS-CD8^+^ T cells, corroborating previous studies demonstrating that anti-insulin B cells, despite being tolerant to antigen, are effective antigen-presenting cells (APCs) [11].

### Selective recruitment of anti-insulin B cells to pancreatic islets

**Fig. 2:**
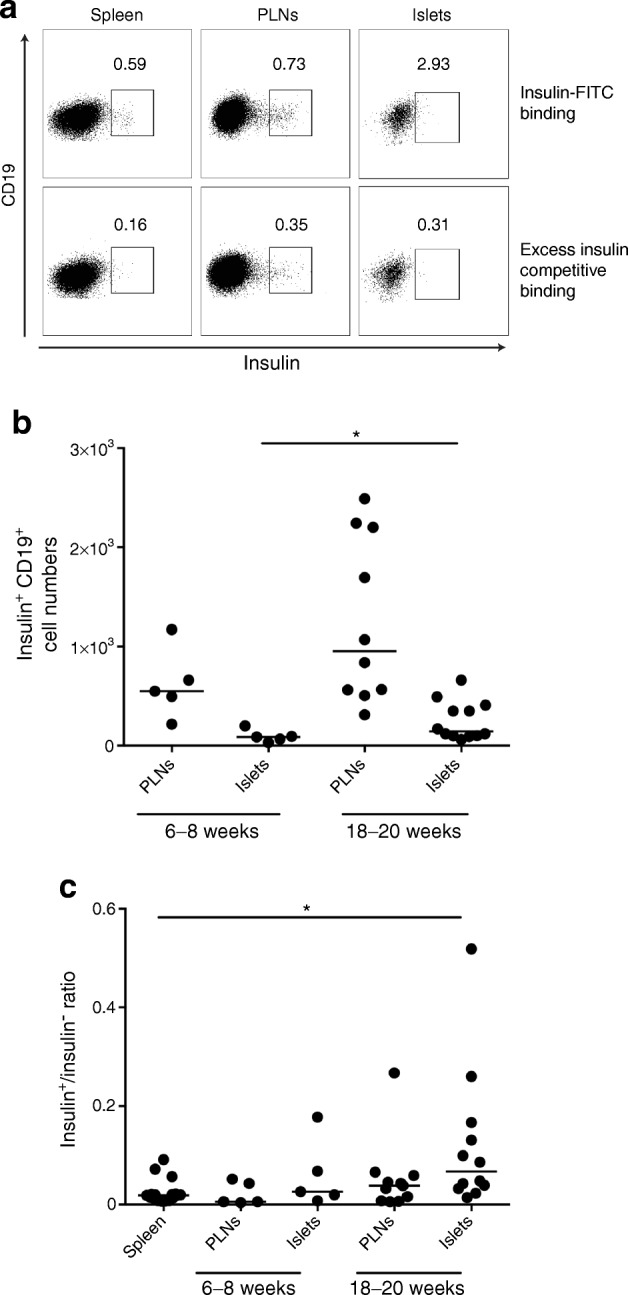
Anti-insulin B cells are selectively recruited to the pancreatic islets. PLNs and pancreatic islets were analysed for the frequency of insulin-positive B cells in VH125.hCD20/NOD mice aged 6–8 weeks (*n* = 5 mice) and 18–20 weeks (*n* = 8 mice [PLNs]; *n* = 12 mice [islets]). Cells were gated on live CD3^−^CD19^+^ populations. (**a**) Flow cytometric plots showing insulin staining in spleen, PLNs and pancreatic islets and competitive binding assay with excess (20×) unlabelled insulin. (**b**) Numbers of insulin-positive B cells from mice aged 6–8 weeks and 18–20 weeks from both PLNs and pancreatic islets. (**c**) Ratio of insulin-positive to insulin-negative B cells in PLNs and pancreatic islets in mice of different ages, compared with spleen (pooled ages, *n* = 17 mice). Horizontal lines represent median values. Data are representative of at least two independent experiments. *p < 0.05 (one-way ANOVA)

We studied anti-insulin B cells in PLNs and pancreatic islets from VH125.hCD20/NOD mice of different ages (Fig. 2). To demonstrate specific binding to insulin, we performed a competitive binding assay [7]. B cells from spleen, PLNs and pancreatic islets were stained with insulin–FITC in the presence of human insulin (Fig. 2a). Successful inhibition of insulin binding on B cells was found in all tissues (ESM Fig. 2b); there was less inhibition in the PLNs (50%), in keeping with insulin-binding B cells in the NOD PLNs having different binding specificities [25]. We ensured that insulin BCR staining was not due to increased insulin receptors on B cells (ESM Fig. 2c), as anti-insulin receptor staining (CD220) did not compete with insulin–FITC detection.

Next, we examined mice with a fully mature B cell repertoire developing early insulitis (aged 6–8 weeks old) and long-established insulitis (18–20 weeks old). We demonstrated a significantly increased frequency of anti-insulin B cells in pancreatic islets (*p* < 0.05) and a non-significant increase in the PLNs (*p* = 0.109); this appeared to coincide with disease progression (Fig. 2b). We compared the number of anti-insulin B cells in the PLNs and islets with numbers of B cells that were not specific for insulin and showed selective recruitment of anti-insulin B cells to the islets (spleen vs islets, *p* < 0.05), whereas the absolute number of anti-insulin B cells was unchanged in the spleen (Fig. 2c). These data suggested that anti-insulin B cells are recruited to the target tissue during beta cell destruction. This was also shown recently [26] and supports the notion that anti-insulin B cells contribute to type 1 diabetes [7].

### Established pancreatic islet B cells are heterogeneous early-differentiated plasma-cell populations

**Fig. 3:**
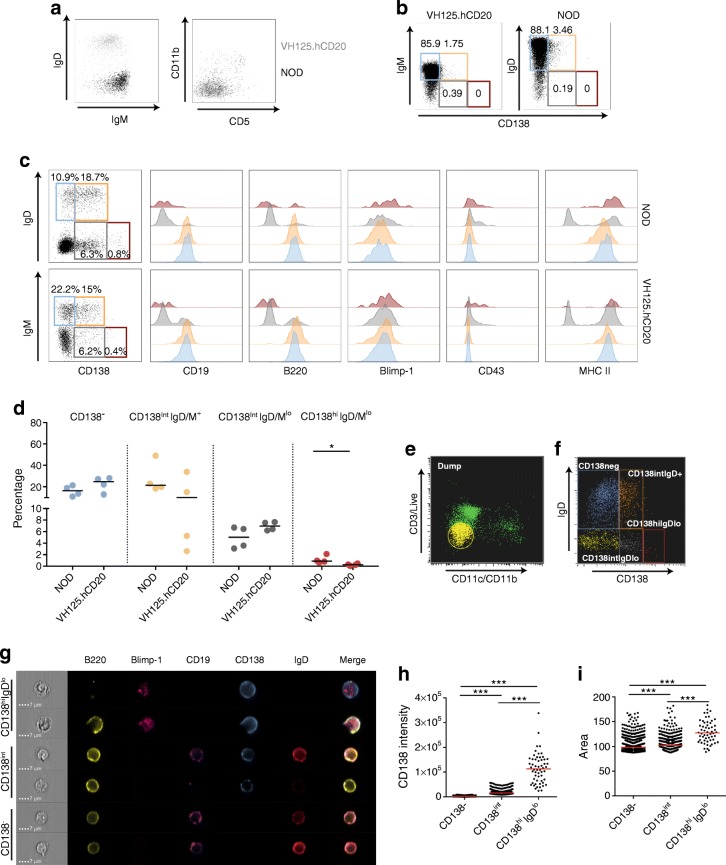
Established pancreatic islet B cell infiltration is enriched for CD138 but few of the cells express Blimp-1. Pancreatic islets from groups (*n* = 2 or 3) of NOD and VH125.hCD20/NOD (VH125.hCD20) mice were pooled, and B cells were analysed by flow cytometry. (**a**) Representative flow plots to show islet B cells expressing IgD, IgM, CD11b and CD5 from NOD mice (black) and VH125.hCD20/NOD mice (grey), gated on live CD3^−^CD19^+^. (**b**–**d**) Gating on live CD3^−^CD11b^−^CD11c^−^ revealed four different populations based on CD138 and IgD/M expression: CD138^−^ (blue gate); CD138^int^IgD/M^+^ (orange gate); CD138^int^IgD/M^lo^ (grey gate) and CD138^hi^IgD/M^lo^ (red gate). Representative flow cytometry plots on splenocytes (**b**) and pancreatic islets (**c**) from NOD and VH125.hCD20/NOD mice; histograms reveal various surface markers and Blimp-1 transcription factor expression on each population. Overall percentages of different CD138 and IgD/M populations in NOD and VH125.hCD20/NOD mice are shown in (**d**). *p < 0.05 (Mann–Whitney *U* test), *n* = 4 groups. (**e**, **f**) Scatter gating for imaging flow cytometry, showing ‘dump’ channel (yellow) of live CD3^−^CD11b^−^CD11c^−^ gate (**e**) and CD138^+^ and IgD^+^ NOD islet populations (**f**). In (**f**): CD138^−^, blue; CD138^int^IgD^+^, orange; CD138^int^IgD^lo^, grey; CD138^hi^IgD^lo^, red. (**g**) Representative images of single cells from CD138^−^, CD138^int^ and CD138^hi^IgD^lo^ NOD islet B cell populations. Scale bars, 7 μm. (**h**, **i**) CD138 intensity (**h**) and cell size measured by area (**i**) on CD138^−^, CD138^int^ and CD138^hi^IgD^lo^ subsets (*n* = 2 groups). Data are representative of at least two independent experiments. Horizontal lines represent median values. ***p < 0.001 (one-way ANOVA)

To investigate total islet-infiltrating B cell population in our transgenic model, we assessed islet B cells from VH125.hCD20/NOD and NOD mice aged 12–20 weeks (Fig. 3). B cells from VH125.hCD20/NOD mice were IgM^+^IgD^−^, whereas in wild-type NOD islets, B cells were IgM^low^IgD^+^ cells (Fig. 3a). During established insulitis, B cells become CD5 negative [12, 14] and upregulate CD138 [27]. We observed few CD11b^+^ or CD5^+^ cells in both NOD and VH125.hCD20/NOD mice, confirming that few B1a B cells were present during insulitis (Fig. 3a).

We defined four subpopulations based on CD138 and IgD/IgM (for gating controls see ESM Fig. 3) expression (IgM on VH125.hCD20/NOD B cells, as the VH125 transgene is IgM and VH125.hCD20/NOD mice do not express IgD). The percentage of splenic B cells (Fig. 3b) expressing CD138 was less than in islet B cells (Fig. 3c) in both NOD and VH125.hCD20/NOD mice. We detected more CD138^+^ B cells in NOD mouse islets compared with VH125.hCD20/NOD mouse islets, although the difference was not statistically significant (*p* = 0.09) (Fig. 3c,d). We observed few plasma cells, defined by high expression of CD138 and loss of IgD/M (Fig. 3c, red gate). CD138^hi^IgD/M^lo^ cells expressed Blimp-1, a key transcription factor for plasma-cell differentiation [28], displayed increased CD43 expression [29] and expressed MHC II molecules (Fig. 3c, red histograms). Although proportions were small, significantly fewer CD138^hi^IgD/M^lo^ B cells were found in VH125.hCD20/NOD islets, compared with NOD islets (Fig. 3d, *p* < 0.05). CD138^int^IgD/M^+^ cells had a similar profile to the CD138^−^ population (Fig. 3c, orange gate vs blue gate).

Single-cell analysis, using imaging flow cytometry, was performed in NOD mice to confirm our observations (Fig. 3e–i). The CD138^int^ cells (Fig. 3f, orange/grey gate) were a heterogeneous population that still expressed B220^+^ but had a reduced (or had lost) CD19 and IgD expression (Fig. 3g). We demonstrated that CD138^hi^IgD^lo^ cells (Fig. 3f, red gate,) expressed Blimp-1, although even in this small population, heterogeneity was visible, as not all cells had lost B220 (Fig. 3g). CD138^int^ and CD138^hi^ populations displayed significantly increased cell size (measured by area), compared with CD138^−^ populations (Fig. 3i). We showed a larger expanse of cytoplasm synonymous with plasma cells, by bright-field imaging. These findings indicated that some B cells, upon entering pancreatic islets, progress into the plasma-cell pathway, although most exist as heterogeneous early-differentiated plasma-cell populations.

### Anti-insulin B cells in pancreatic tissue are enriched in the CD138^int^ subset

**Fig. 4:**
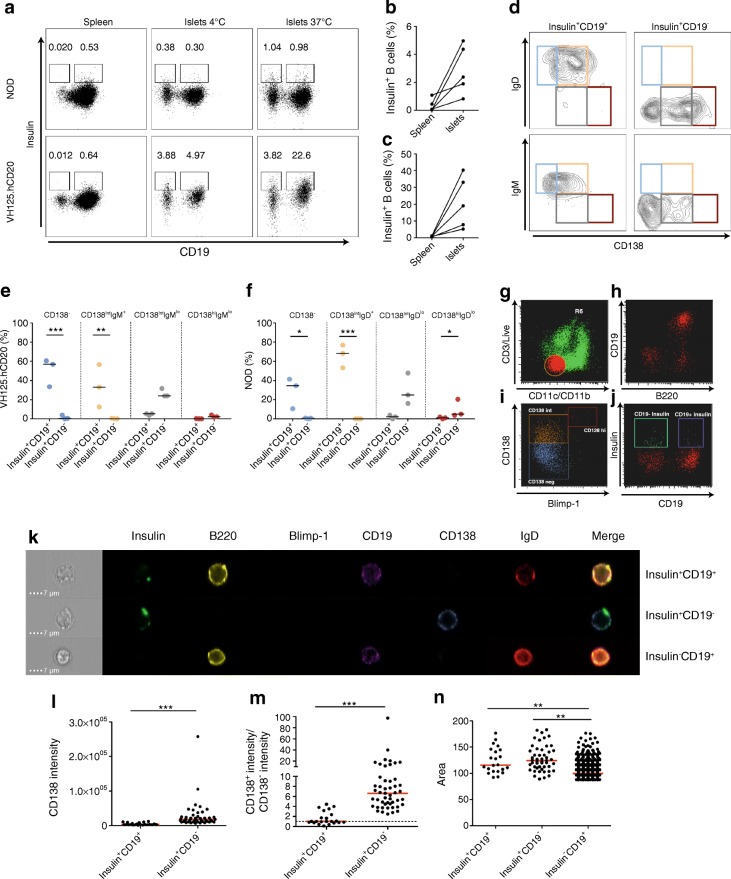
Anti-insulin islet B cells are enriched in the CD138^int^ subset. Pancreatic islets from groups (*n* = 2 or 3) of NOD and VH125.hCD20/NOD (VH125.hCD20) mice were pooled and insulin^+^ B cells were analysed by flow cytometry. (**a**) Representative flow plots showing insulin-positive B cells in spleen and islets, either stained at 4°C or 37°C, against CD19 expression in NOD and VH125.hCD20/NOD mice. (**b**, **c**) Line graphs represent percentages of insulin-positive B cells from spleens and islet that were detected by staining at 37°C in NOD (**b**) and VH125.hCD20/NOD mice (**c**) (*n* = 4 groups). (**d**) Four different populations express different combinations of CD138 and IgD in NOD mice and IgM in VH125.hCD20/NOD mice: CD138^−^IgD^+^/IgM^+^ (blue); CD138^int^IgD^+^/IgM^+^ (orange); CD138^int^IgD^lo^/IgM^lo^ (grey) and CD138^hi^IgD^lo^/IgM^lo^ (red). (**e**, **f**) Percentage of insulin^+^CD19^+^ and insulin^+^CD19^−^ enrichment categorised by CD138 and IgM in VH125.hCD20 mice (**e**) and IgD subsets in NOD mice (**f**) (*n* = 3 groups). *p < 0.05, **p < 0.01 and ***p < 0.001 (two-way ANOVA). (**g–j**) Scatter gating for imaging flow cytometry on NOD islets that were pulsed with insulin–FITC at 37°C, showing: ‘dump’ channel (red with orange outline) of live CD3^−^CD11b^−^CD11c^−^ gate (**g**); B220 and CD19 expression on CD3^−^CD11b^−^CD11c^−^ cells (red) (**h**); CD138 and Blimp-1 defined populations (CD138^−^, blue; CD138^int^, orange; CD138^hi^, red) (**i**); and CD19^−^ (green) and CD19^+^ (purple) insulin^+^ B cells (**j**). (**k**) Representative single-cell images from NOD islet insulin^+^CD19^+^, insulin^+^CD19^−^ and insulin^−^CD19^+^ B cell populations. Scale bars, 7 μm. (**l**, **m**) CD138 staining intensity (**l**) and CD138 intensity/CD138^−^ intensity ratio (**m**) on insulin^+^CD19^+^ and insulin^+^CD19^−^ B cell populations. ***p < 0.001 (Mann–Whitney *U* test). (**n**) Cell size measured by area on insulin^+/−^ B cell populations (*n* = 2 groups). **p < 0.01 and ***p < 0.001 (one-way ANOVA). Data are representative of at least two independent experiments. Horizontal red and black lines represent median values

We next questioned whether islet anti-insulin B cells were present in the compartments shown in Fig. 3. Islet anti-insulin B cells may already have endogenous insulin bound to their BCRs. To ensure that we were detecting all anti-insulin B cells, islets from groups of mice were pooled and incubated with insulin–FITC at 37°C. We observed an increased frequency of anti-insulin B cells compared with islets stained at 4°C (Fig. 4a). Two insulin-specific populations were identified: insulin^+^CD19^+^ and insulin^+^CD19^−^ B cells (Fig. 4a). We detected increased proportions of insulin^+^CD19^+^ B cells in VH125.hCD20/NOD mice compared with NOD mice, with variability in both strains (Fig. 4b,c). We also demonstrated increased insulin^+^CD19^+^ cells in pancreatic islets, compared with spleen, in both strains (Fig. 4b,c), supporting earlier observations (Fig. 2). Competitive binding experiments confirmed that both insulin^+^CD19^+^ and insulin^+^CD19^−^ populations were insulin specific (ESM Fig. 2a,b). To study expression of CD138 and IgD/IgM, we used the gating strategy shown in Fig. 3. Insulin^+^CD19^+^ and insulin^+^CD19^−^ cells expressed intermediate CD138 in VH125.hCD20/NOD and NOD strains (Fig. 4d–f). We found a higher proportion of plasma cells (CD138^hi^IgM/D^lo^) among the insulin^+^CD19^−^ population. Differences between the mice were highlighted by a higher proportion of the insulin^+^CD19^+^ cells in the CD138^−^ fraction (Fig. 4d, blue gate, *p* < 0.001) and a lower proportion in the CD138^int^ fraction (Fig. 4d, orange gate, *p* < 0.01) in the VH125.hCD20/NOD mice. The converse relationship was found in NOD mice (Fig. 4d; blue gate, *p* < 0.05; orange gate, *p* < 0.001).

To evaluate cell morphology, we used single-cell imaging. Spleens and pooled islets from VH125.hCD20/NOD mice (ESM Fig. 4) were analysed alongside pooled NOD mouse islets. Gating on live CD3^−^CD11c^−^CD11b^−^ revealed anti-insulin B cell populations divided by their CD19 expression (Fig. 4g–j). We confirmed that anti-insulin B cells were enriched in the CD138 subset. Insulin^+^CD19^−^ cells also displayed loss of IgD and little expression of Blimp-1 (Fig. 4k), supporting the observation that very few cells in the CD138^hi^IgD^lo^ population bound insulin. We demonstrated that CD138 staining intensity was significantly increased in the insulin^+^CD19^−^ B cell population, compared with the CD19^+^ population (Fig. 4l). However, some insulin^+^CD19^+^ B cells displayed increased CD138 expression, when compared with the intensity of the CD138^−^ B cell subset (Fig. 4m, dotted line), but insulin^+^CD19^−^ B cells displayed significantly greater expression (Fig. 4l). Finally, cell size analysis revealed that both of the insulin-positive populations were larger compared with CD19^+^ insulin-negative B cells, indicating that these cells were activated and blasting (Fig. 4n).

### Anti-insulin B cells are recruited to pancreatic islets after anti-CD20 treatment

B cell depletion therapy has been successful in delaying the onset of diabetes [2, 3, 30]. However, the effect of global B cell depletion on anti-insulin B cells has not been studied. We confirmed that expression of VH125 had no effect on hCD20 expression (ESM Fig. 5a) and that hCD20-expressing B cells were present in the tissues examined (ESM Fig. 5b). We treated groups of 6- to 8-week-old VH125.hCD20/NOD mice with anti-CD20 mAb, and analysed spleen and PLN tissue for anti-insulin B cells after treatment. Expression of hCD20 was similar in anti-insulin and non-insulin-binding B cells, with successful targeting of both populations in spleen (ESM Fig. 5c) and PLNs at 24 h after treatment (ESM Fig. 5d). In agreement with previous studies [31, 32], expression of murine CD20 was parallel with the expression of human CD20 (ESM Fig. 5e). These results suggest that autoreactive B cells in the periphery are successfully depleted and not spared by anti-CD20 treatment.

As anti-insulin B cells are altered upon entry into islets, we investigated whether these cells would be targeted. We confirmed that IgM^+^ B cells were targeted by treatment (ESM Fig. 6a), which we determined was a result of hCD20 expression (ESM Fig. 6b). CD19^+^insulin^+^ B cells were successfully depleted, although cell numbers were low, making it difficult to determine statistical significance (ESM Fig. 6c,e). However, we observed hCD20 expression on CD19^+^insulin^+^ B cells (ESM Fig. 6d), indicating that anti-insulin B cells are targeted in pancreatic islets. Again, we found that hCD20 expression was parallel with murine CD20 on islet B cells (ESM Fig. 6b,d). Conversely, hCD20 was not expressed on insulin^+^CD19^−^ B cells, indicating that these cells would be spared by anti-CD20 treatment (ESM Fig. 6d). During the 12 week observation period, 48% of the control anti-CD20 antibody-treated mice became diabetic compared with 15% of the 2H7-treated mice.

**Fig. 5:**
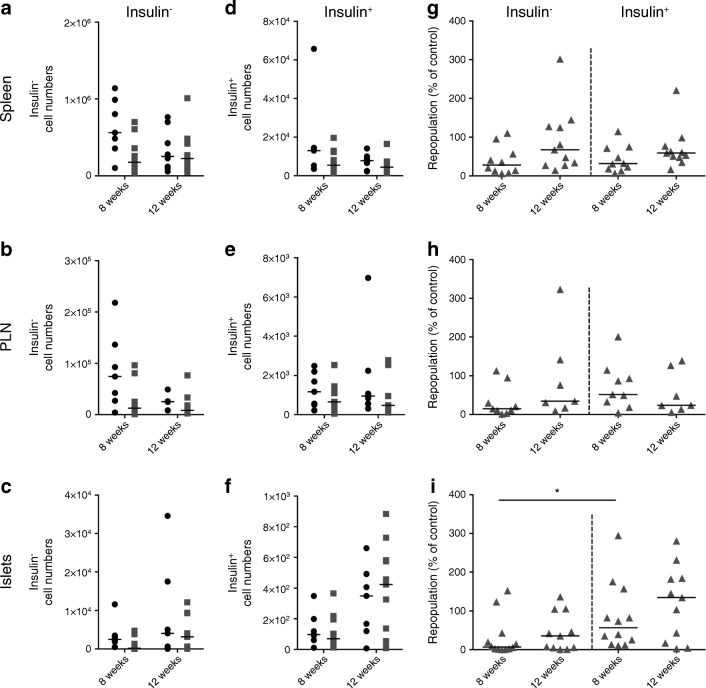
Anti-insulin B cells repopulate pancreatic islets more rapidly than insulin-negative B cells after anti-CD20 treatment. Groups of VH125.hCD20/NOD mice, aged 6–8 weeks old, were injected with 2H7 anti-CD20 or IgG isotype control. Spleen, PLNs and pancreatic islets were analysed for insulin-positive and insulin-negative B cells at 8 weeks and 12 weeks post depletion by flow cytometry. (**a**–**f**) No. of cells from IgG control-treated (black circles) and 2H7-treated (grey squares) mice for insulin-negative B cells (**a**–**c**) and insulin-positive B cells (**d**–**f**) from spleen (**a**, **d**) PLNs (**b**, **e**) and islets (**c**, **f**). (**g**–**i**) Percentage of B cells repopulated at 8 and 12 weeks after treatment from spleen (**g**), PLNs (**h**) and islets (**i**) of mice shown in (**a**–**f**). Percentages were calculated as individual numbers from each 2H7-treated mouse / mean number from all IgG control antibody-treated mice. Horizontal lines represent the median value. Data represent three independent experiments. At 8 weeks, *n* = 7 (spleen), *n* = 7 (PLNs) and *n* = 9 (islets) for control IgG-treated mice and *n* = 10 (spleen), *n* = 9 (PLNs) and *n* = 12 (islets) for 2H7-treated mice. At 12 weeks, *n* = 8 (spleen), *n* = 6 (PLNs) and *n* = 7 (islets) for control IgG and *n* = 11 (spleen), *n* = 7 (PLNs) and *n* = 11 (islets) for 2H7-treated mice. *p < 0.05 (one-way ANOVA)

To investigate the repopulation of anti-insulin B cells, we examined CD19^+^insulin^+^ and CD19^+^insulin^−^ cells at 8 and 12 weeks after depletion (Fig. 5). In hCD20/NOD mice, B cells repopulate peripheral tissues by 12 weeks post treatment [2, 19]. We found repopulation dynamics were similar for anti-insulin B cells in spleen (Fig. 5a–g) and PLNs in VH125.hCD20/NOD mice (Fig. 5b–h). However, we observed that anti-insulin B cells repopulated earlier than non-insulin B cells in pancreatic islets (Fig. 5i).

**Fig. 6:**
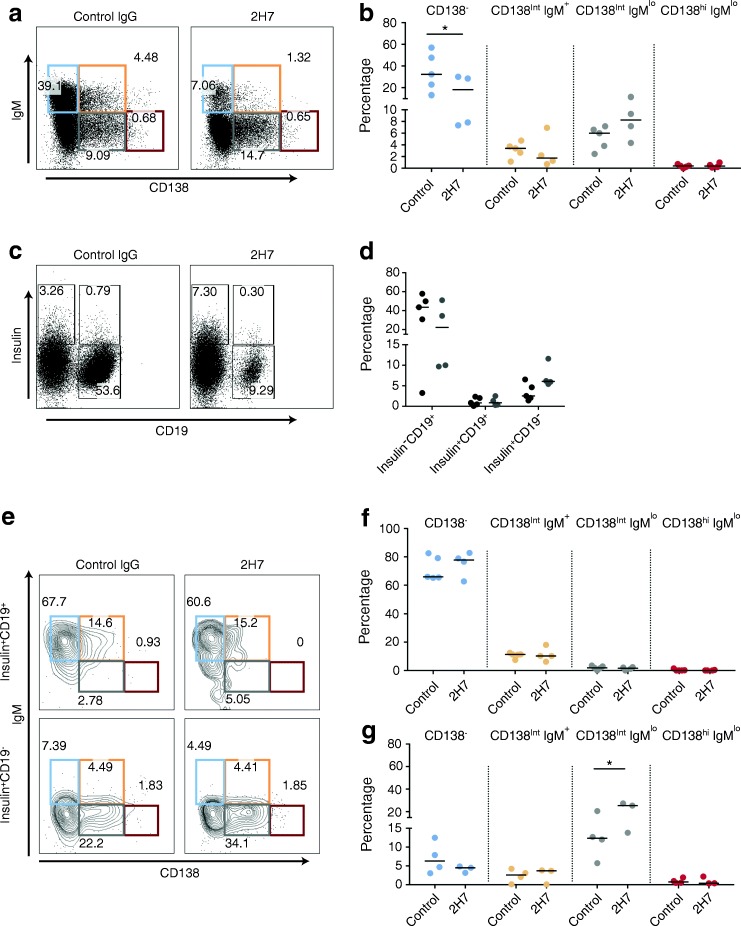
CD138^int^ anti-insulin B cells are enriched in pancreatic islets after anti-CD20 treatment. Groups of 6- to 8-week-old VH125.hCD20/NOD mice were injected with 2H7 anti-CD20 or IgG isotype control. Groups of mice (*n* = 2 or 3 per group) were pooled and insulin^+^ B cells from pancreatic islets were analysed for four different populations based on CD138 expression: CD138^−^ (blue); CD138^int^IgM^+^ (orange); CD138^int^IgM^lo^ (grey) and CD138^hi^IgM^lo^ (red). (**a**, **b**) Representative flow plots showing gating on live CD3^−^CD11b^−^CD11c^−^ (**a**) and graph showing the overall percentages of the four different populations (**b**). (**c**, **d**) Representative flow plots showing insulin^−^CD19^+^, insulin^+^CD19^+^ and insulin^+^CD19^−^ cells (**c**) and graph showing the overall percentages of these cells (**d**); 2H7 (black circles), IgG (grey circles). (**e**) Representative flow plots showing CD138 and IgM expression in insulin^+^CD19^+^ and insulin^+^CD19^−^ cells. (**f**, **g**) Graphs showing CD138 and IgM populations on insulin^+^CD19^+^ (**f**) and insulin^+^CD19^−^ cells (**g**) (*n* = 5 groups for control IgG treatment; *n* = 4 groups for 2H7 treatment). Horizontal lines represent the median values. Data represent two independent experiments. *p < 0.05 (one-way ANOVA)

To analyse anti-insulin B cells further, we pooled pancreases to gain more cells for analysis (Fig. 6). Considering that anti-insulin B cells are enriched in the CD138^int^ fractions (Fig. 4), we analysed IgM and CD138 subpopulations after 12 weeks of treatment. Islet B cells expressing IgM were reduced in 2H7-treated mice (Fig. 6a,b), although interestingly an increase in the CD138^int^IgM^lo^ fraction was observed (Fig. 6b). In line with this, we observed an increase in the percentage of insulin^+^CD19^−^ cells (Fig. 6c,d), which were significantly enriched in the CD138^int^IgM^lo^ fraction (Fig. 6e–g) after anti-CD20 treatment.

## Discussion

In this study, we describe novel anti-insulin B cell populations that reside in the pancreatic islets during type 1 diabetes development. Anti-insulin B cells are selectively recruited to pancreatic tissue during diabetes progression and upon entry these cells assume a unique CD138^int^ phenotype. Furthermore, for the first time, we show that during global B cell depletion, anti-insulin B cells are not spared but are targeted in VH125.hCD20/NOD mice and, importantly, we have identified a key pattern of repopulation after resetting the B cell repertoire. This work highlights the need to further understand the dynamics of anti-insulin B cells.

Previous work performed in VH125Tg and V_H_125^SD^ NOD mouse models supports the notion that anti-insulin B cells are functionally active, respond to mitogens and are effective APCs despite their tolerant state [7, 11, 33]. Here, we show that anti-insulin B cells from our VH125.hCD20/NOD mouse model successfully present antigen to insulin-specific CD8 T cells. Consequently, anti-insulin B cells not only present to CD4 T cells [11] but also successfully present to pathogenic insulin-specific CD8 T cells.

Insulin-reactive B cells escape immune tolerance in mice susceptible to type 1 diabetes [8]. Indeed, we show that anti-insulin B cells are selectively recruited to pancreatic tissue in VH125.hCD20/NOD mice. Our data supports a previous study, using a different method of detection (insulin-specific mAb123-biotin), showing increased insulin-binding B cells in the pancreas of VH125Tg/NOD mice [15]. While we have not addressed whether anti-insulin B cells are specifically recruited via a chemokine receptor, it is conceivable that anti-insulin B cells express high levels of C-X-C motif chemokine receptor 3 (CXCR3), a chemokine receptor involved in recruitment of lymphocytes in autoimmunity [34, 35]. High levels of CXCR3 are expressed on other autoreactive B cell subsets [36] and CXCR3 is involved in localisation of plasma cells [37]. Further investigation is required to fully understand whether the enrichment observed is a result of recruitment or retention.

We reveal that anti-insulin B cells are unique, although heterogeneous, in pancreatic islets, with enrichment of CD138 and loss of their naive BCR isotypes (IgM/IgD). This suggests that some B cells enter the plasma-cell differentiation pathway. Although the expression of CD138 on pancreatic islet B cells has been described previously [27], and more recently on insulin-binding B cells in VH125.NOD mice [26], this is the first time the heterogeneity has been reported. Furthermore, CD138 intermediate expression coupled with a lack of Blimp-1 demonstrates that islet B cells are not terminally differentiated. This does not exclude the possibility that CD138^hi^ B cells have previously expressed an insulin-specific BCR but lost the BCR on terminal differentiation, [38, 39] so are unable to detect and bind insulin. CD138^hi^IgD^lo^ B cells remain MHC II positive. However, as MHC II is lost on terminally differentiated B cells, these islet CD138^hi^IgD^lo^ B cells may also represent an early differentiated plasma-cell type [37] or a short-lived plasma-cell [40]. While loss of BCR expression would render B cells unresponsive to antigen, expression of MHC II would still allow antigen presentation in an inflammatory microenvironment, possibly driving beta cell destruction. Intermediate expression of CD138 is reminiscent of autoreactive anti-sm (ribonucleoprotein Smith) B cells described in the spleen of autoimmune mice [41]. However, some CD138^int^ anti-insulin B cells that are still IgD competent may resemble IgM^low^IgD^high^ mature follicular B cells, a unique splenic follicular zone subset [42]. It is clear CD138^int^ and CD138^hi^ are distinct populations, and their relationship and role in auto-inflammation is yet to be defined.

We note that wild-type NOD islet B cells resemble the B_ND_ (anergic naive) compartment in peripheral blood of humans, which are also IgM^low^IgD^+^ in phenotype. This anergic B_ND_ compartment is enriched with a pool of autoreactive B cells [43]. However, these B_ND_ cells are lost in individuals with newly diagnosed type 1 diabetes [24] for reasons that are currently unclear. Our data support the notion that these B cells may have relocated to pancreatic islets and are thus lost from peripheral blood. Interestingly, co-expression of IgD and IgM promotes accumulation of anergic B cells and increased CD138 expression is associated with reduced IgM [44]. Furthermore, a loss of IgD expression on B cells induces amplified CD138 expression [44], possibly reflecting the response of anti-insulin B cells entering pancreatic tissue. In humans, CD138^+^ B cells have been detected in islets that are positive for CD20^+^ B cells in five out of 29 individuals studied [45, 46]. This suggests either that there are similar migration patterns in human pancreatic islets or that B cells change in situ, the latter being consonant with our observations.

Our work in this diabetes model is consistent with earlier studies in arthritis [32] showing successful depletion of peripheral autoreactive B cells using an hCD20 transgenic system. While a previous study suggested that all B cells downregulate CD20 upon islet entry [27], we show this is not the case and that a heterogeneous population exists, which includes CD20^+^ B cells. This disparity may be due to the different anti-CD20 mAb used. We detected some anti-insulin B cells that had lost CD20 expression and were spared from anti-CD20 treatment. However, this population is small and diabetes is delayed in a large proportion of mice and hence anti-CD20 treatment is clearly beneficial, at least temporarily.

The early insulin-positive B cell recruitment to pancreatic islets after anti-CD20 treatment is an important observation in the therapeutic use of B cell depletion. We demonstrate that insulin^+^CD19^−^ B cells are increased in the pancreatic environment upon repopulation, indicating that during repopulation, pancreas-infiltrating anti-insulin B cells are recruited earlier and enter the plasma-cell differentiation pathway more rapidly. Alternatively, B cells (developing B cells [47]) already expressing CD138 in the bone marrow exit prematurely after anti-CD20 treatment and track to pancreatic islets. Anti-CD20 treatment alters the islet microenvironment [19] and may allow CD19^−^ anti-insulin B cells to proliferate in situ or receive increased survival signals. B cell activating factor (BAFF) improves the survival rate of autoreactive B cells [48] and increased BAFF levels are seen in individuals after B cell depletion therapy [49, 50]. Factors such as BAFF may influence the return of the autoreactive B cell repertoire and allow anti-insulin B cells to populate pancreatic tissue following anti-CD20 treatment.

The function of both CD138^int^ populations is of importance, particularly the enriched insulin^+^CD19^−^ observed after anti-CD20 treatment. These cells may be functionally altered and may change the pancreatic environment. Whether early recruitment of anti-insulin B cells to the pancreas after global B cell depletion contributes to disease persistence or, alternatively, may in some way participate in the delay of diabetes needs to be addressed. This latter possibility is of particular interest, as recently CD138 (syndecan 1) has been identified as a hallmark of anergy [44]. It should also be considered that the insulin^+^ B cells we have defined have opposing roles. We acknowledge that while we would like to test these possibilities, functional assays on such small populations is highly technically challenging and would require many pancreas samples to be pooled from large numbers of experimental animals for each group, rendering this unfeasible with current technology. However, this would be an important investigation for the future. Nevertheless, our study highlights a unique phenotype of islet-infiltrating B cells, emphasising the need for greater understanding of autoreactive B cells and how they promote the development of type 1 diabetes, as well as providing novel insight to help with better design of more effective immunotherapies.

## Electronic supplementary material

ESM Figures (PDF 6331 kb)

## Data Availability

The datasets generated and/or analysed during the current study are available from the corresponding author on reasonable request.

## Abbreviations

APC: Antigen-presenting cell
BAFF: B cell activating factor
BCR: B cell receptor
CXCR3: C-X-C motif chemokine receptor 3
GMFI: Geometric mean fluorescent intensity
hCD20: Human CD20
mAb: Monoclonal antibody
PLN: Pancreatic lymph node
