# Supplementary material for: Phenotypically distinct anti-insulin B cells repopulate pancreatic islets after anti-CD20 treatment in NOD mice
Source: Diabetologia. 2019 Aug 23;62(11):2052–65. doi: 10.1007/s00125-019-04974-y (PMC6805803; doi:10.1007/s00125-019-04974-y)
Supplement: Supplementary file 1 — (PDF 6331 kb) [file 125_2019_4974_MOESM1_ESM.pdf]

ESM Fig. 1

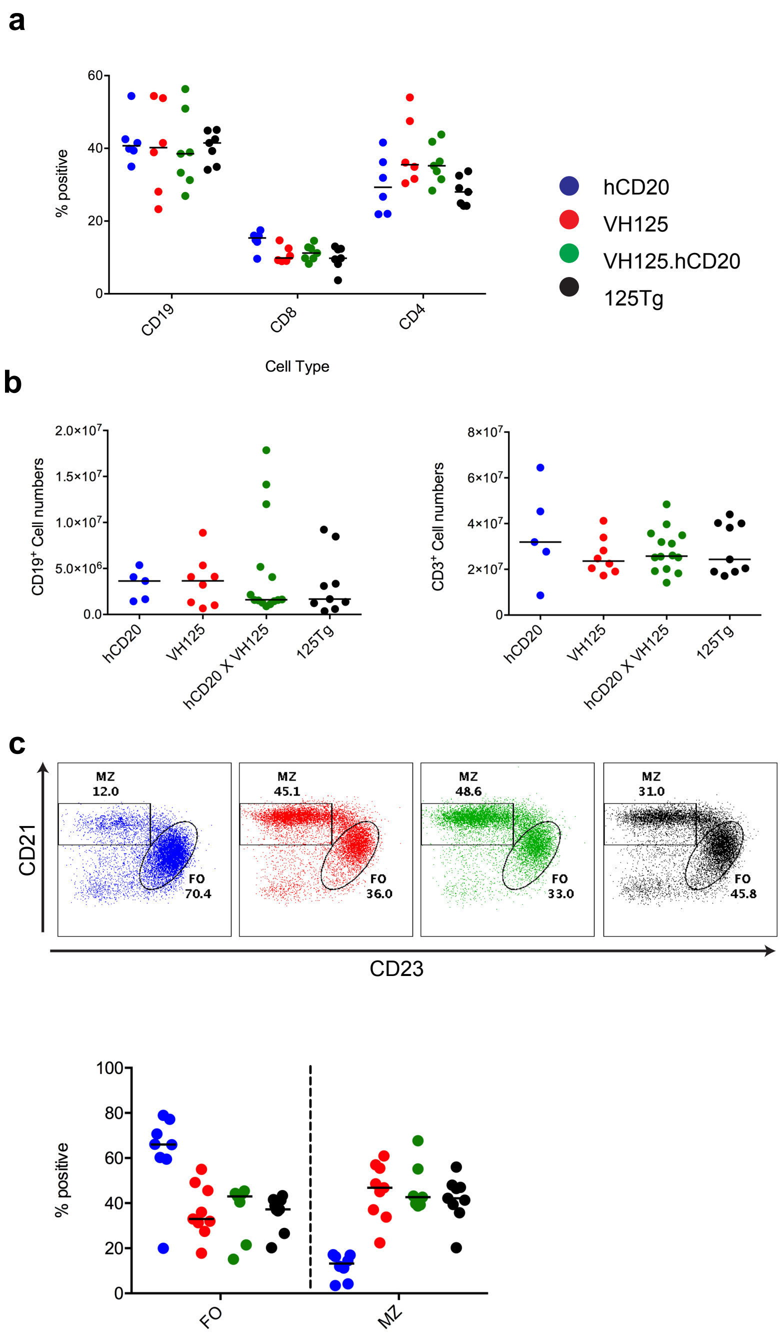

ESM fig. 1. *Characterization of VH125.hCD20/NOD mice.* Spleens of hCD20 (blue), VH125 (red), VH125.hCD20/NOD (green) and 125Tg (black) were analyzed by flow cytometry. (a) Percentage of $CD19^+$ B-cells and $CD8^+$ and $CD4^+$ T ($CD3^+$) cells. (b) Absolute numbers of $CD19^+$ B-cells (left) and $CD3^+$ T-cells (right). (c) Representative flow cytometry plots of splenocytes stained with antibodies against CD21 and CD23 marking follicular (FO, $CD23^{hi}CD21^{lo}$) and marginal (MZ, $CD23^{lo}CD21^{hi}$) zones B-cells (top), and summary graph showing average percentages of the subsets in splenocyte compartments (bottom). Data are representative of 3 independent experiments. Mice analyzed were 6-10 weeks old.

ESM Fig. 2

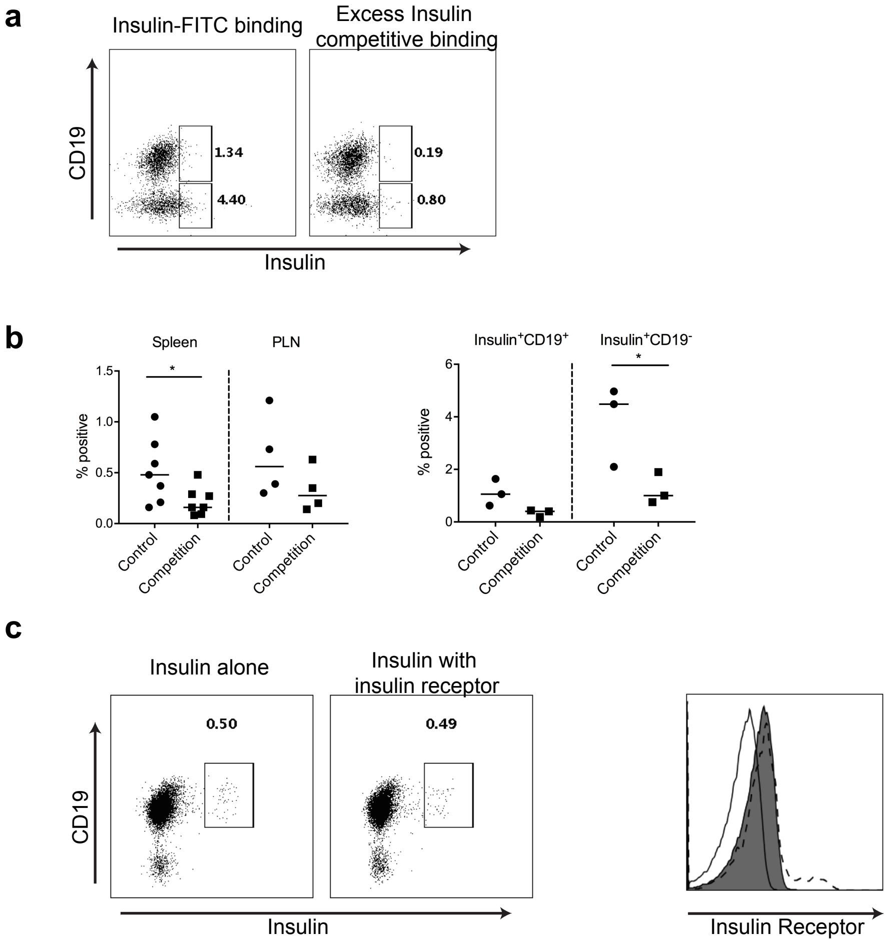

ESM fig. 2. *Insulin competition in VH125.hCD20/NOD mice.* (a) Flow cytometric plots showing anti-insulin staining in pancreatic islets, at 4°C, and competitive binding assay with excess x20 unlabeled insulin. (b) Summary competition data from both spleen and PLN (left) and pancreatic islet populations (right). (c) Representative flow plots from live $CD3^-$ splenocytes with and without insulin receptor antibody (left) and insulin receptor (CD220) positive staining example on B cells (filled histogram) and $CD11c^+CD11b^+$ cells (dashed histogram) and fluorescence minus one (open histogram) (right). *P<0.01; Mann-Whitney U test.

ESM Fig. 3

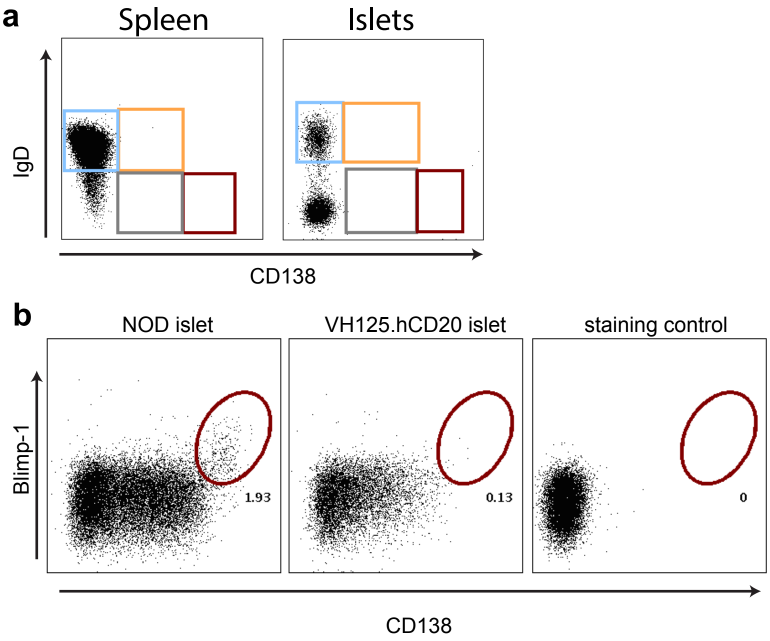

ESM fig. 3. *Gating controls for CD138 populations.* (a) flow cytometric plots showing CD138 fluorescence minus one (FMO) in both spleen and islets to determine CD138^{+} B-cells (b) Gating strategy to define CD138hi B-cell population against Blimp-1 expression in both NOD and VH125.hCD20 pancreatic islets with staining control. Cells were gated on Live CD3^{-}CD11b^{-}CD11c^{-} cells.

ESM Fig. 4

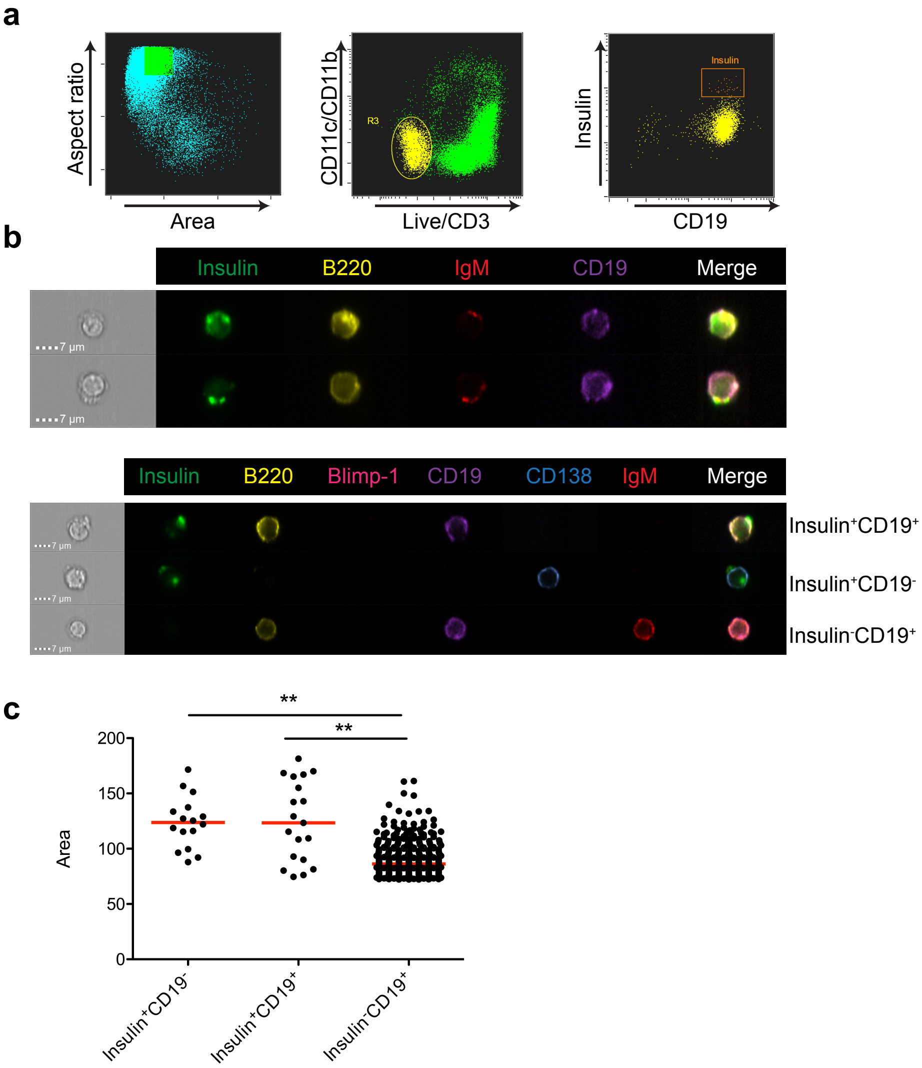

ESM fig. 4. *VH125.hCD20/NOD anti-insulin B-cells identified by imaging flow cytometry.* Spleen and pancreatic islets from VH125.hCD20/NOD mice were pooled and B-cells were analyzed by imaging flow cytometry. (a) Gating strategy showing singlets, live, $CD3^{-}CD11b^{-}CD11c^{-}$ splenocytes that are $CD19^{+}$insulin^{+} (b) Representative single cell images of spleen insulin^{+} B-cells expressing B220, IgM and CD19 (top panels) and islet insulin^{+}CD19^{+}, insulin^{+}CD19^{-} and insulin^{-}CD19^{+} B-cells expressing various surface markers and transcription factor BLIMP-1 (bottom panels). (c) Cell size measured by area on insulin$^{+/-}$ populations. Red lines represent median values. Data are representative of 2 independent of experiments. **$P<0.01$, one way ANOVA.

ESM Fig. 5

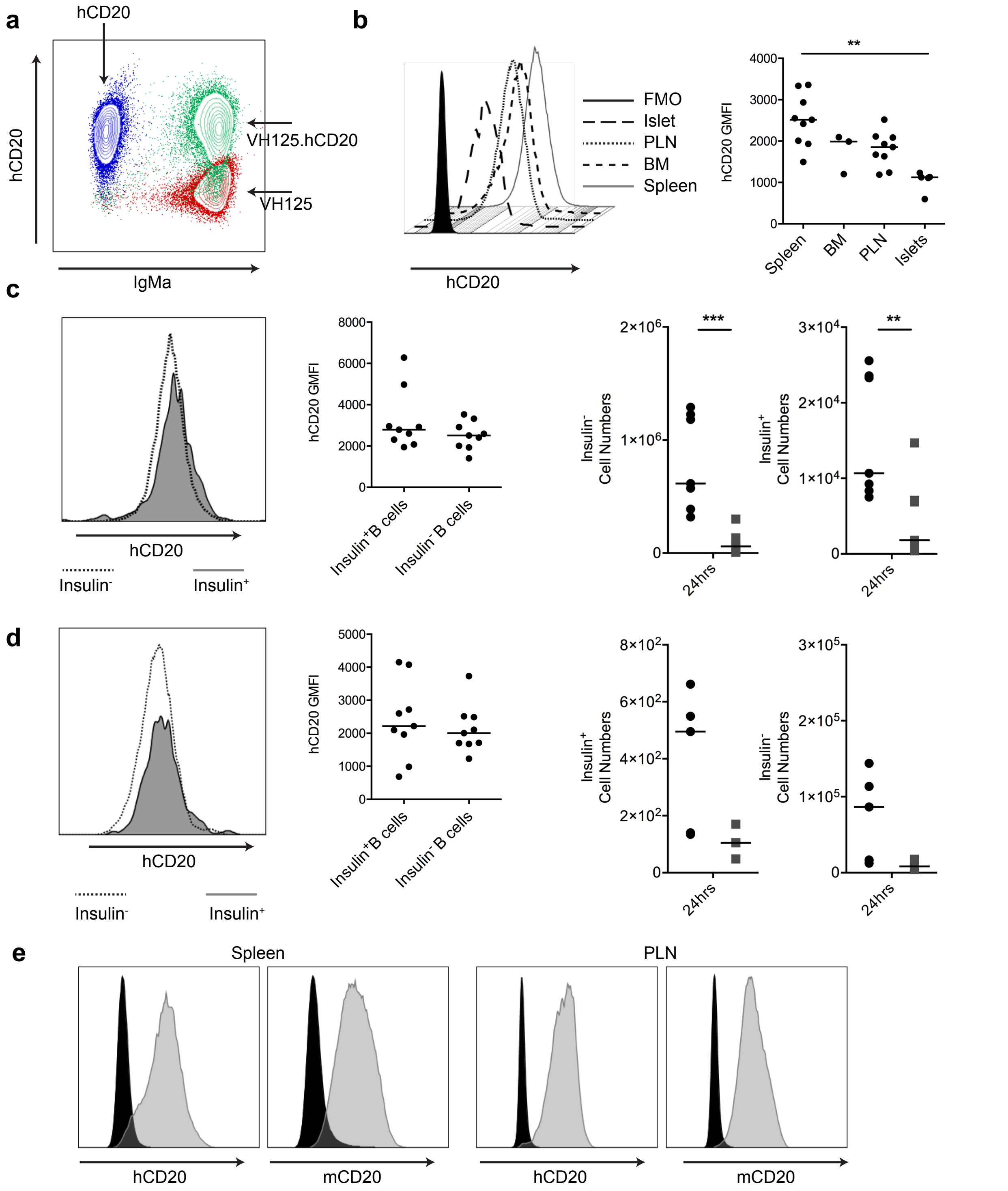

ESM fig. 5. *hCD20 expression in VH125.hCD20/NOD mice.* Spleen, bone marrow (BM), pancreatic lymph nodes (PLN) and pancreatic islets from VH125.hCD20/NOD mice were analyzed for human CD20 (hCD20) and murine CD20 (mCD20) expression. (a) Plot showing IgM^a (allotype of VH125) and hCD20 on VH125.hCD20/NOD mice (green cells), using single transgenic VH125 (red cells) and hCD20 mice (blue cells) as controls. (b) Histogram plot (left) and summary graph (right) showing hCD20 expression by geometric mean fluorescence intensity (GMFI) on $CD19^+IgM^{a+}$ B-cells in spleen, BM, PLN and pancreatic islets. Fluorescence minus one (FMO) control is shown in black, **P<0.01; one-way ANOVA. (c,d) hCD20 expression (left, middle left) and anti-CD20 treatment at 24hrs (right middle, right) on $insulin^+$ and $insulin^-$ $CD19^+IgMa^+$ B-cells in the spleen (c) and PLN (d) **P<0.01, *** P<0.001; Mann-Whitney U test.(e) hCD20 and mCD20 expression on $CD19^+$ $IgMa^+$ B cells (grey histogram) in the spleen (left) and PLN (right), FMO control is shown in black. Data represents at least 2 independent experiments.

ESM Fig. 6

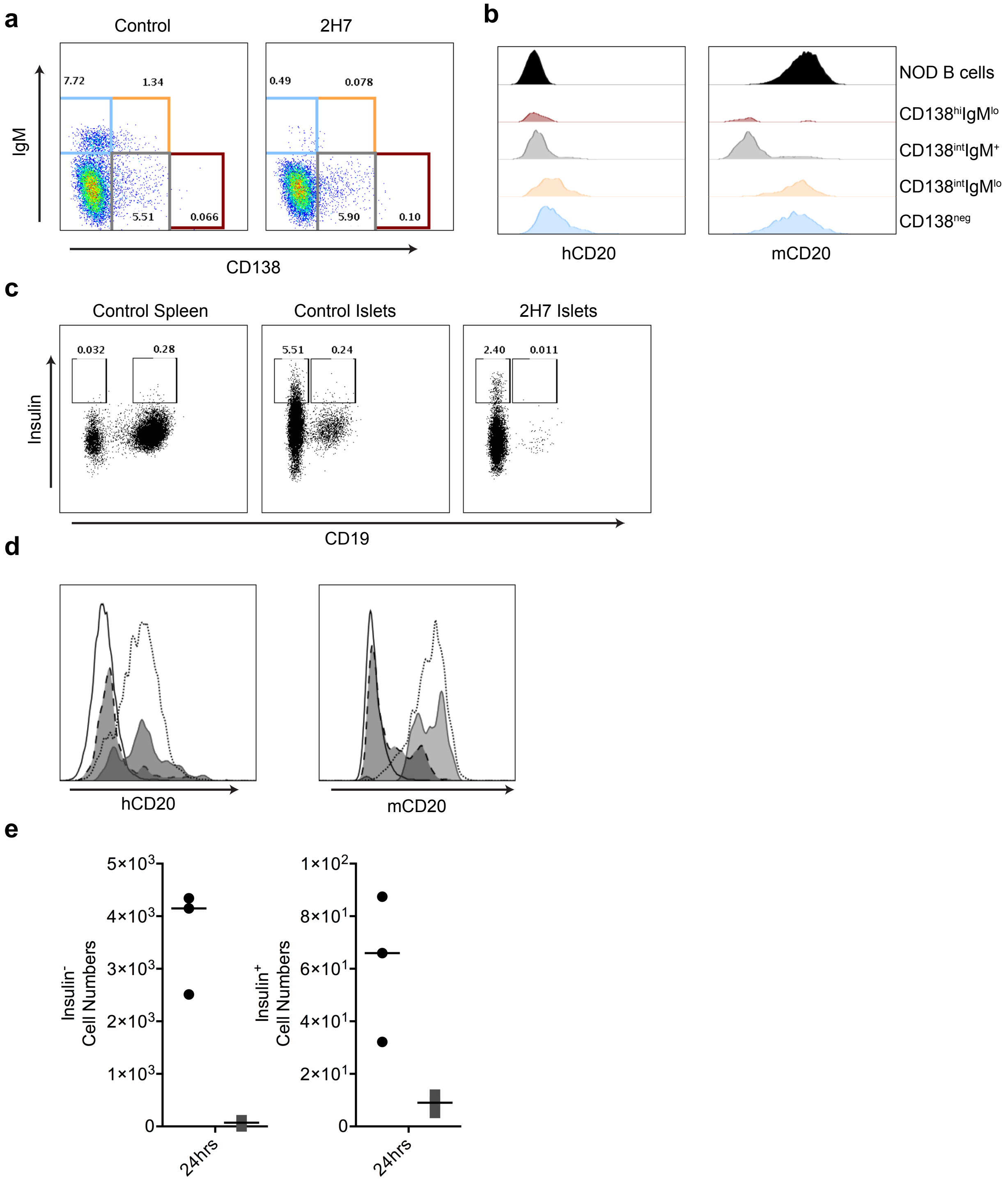

ESM fig. 6. *Pancreatic islet CD19⁺ Insulin⁺ B cells are depleted with anti-CD20 treatment in VH125.hCD20/NOD mice* (a) Flow cytometry plots showing pancreatic islets gated by IgM and CD138 populations 24hrs after treatment with anti-CD20 (2H7). (b) Human and murine CD20 expression on gated populations from control shown in (a), NOD islet $CD19^+$ B cells shown in black filled histogram. (c) Representative flow plots showing $insulin^+CD19^+$ and $insulin^+CD19^-$ B cells from mice treated with control IgG and 2H7. Control spleen (left), control islets (middle) and 2H7 (right). (d) Histograms demonstrating both hCD20 (left) and mCD20 (right) expression on different islet populations, open black line: islet non-B cells, dotted line: $CD19^+$ total B cells; filled black line: $insulin^+CD19^+$; Filled dashed line: $insulin^+CD19^-$ (e) Cell numbers in pancreatic islets taken from control IgG and 2H7 treated mice after 24hrs. Data represent at least one independent experiment.
